# A Novel Green Lithium Oxide Nanoparticle for Adsorption of the Escitalopram Oxalate and *In Vitro* Safety Profile

**DOI:** 10.1021/acsomega.5c02918

**Published:** 2025-09-09

**Authors:** Sthéfany Nunes Loureiro, Leandro Rodrigues Oviedo, Daniel Moro Druzian, Yolice Patrícia Moreno, Giovani Pavoski, Denise Crocce Romano Espinosa, Gabriela Geraldo Sangoi, Alencar Kolinski Machado, William Leonardo Da Silva

**Affiliations:** † Applied Nanomaterials Research Group (GPNAp), 42510 Franciscan University (UFN), Santa Maria 97010-032, RS, Brazil; ‡ Department of Fundamental Chemistry (DQF), Federal University of Pernambuco (UFPE), Recife 50740-560, PE, Brazil; § Polytechnical School of Chemical Engineering, 28133 University of the Sao Paulo (USP), São Paulo 05403-000, SP, Brazil; ∥ Laboratory of Cell Culture and Bioactive Effects, Franciscan University (UFN), Santa Maria 97010-032, RS, Brazil

## Abstract

One of the major environmental and socioeconomic challenges today is the improper disposal of stable pharmaceutical compounds. In this context, this study focuses on the biosynthesis and characterization of lithium oxide nanoparticles (Li_2_O-NPs) using *Mentha arvensis* extract for the adsorption-based removal of the escitalopram oxalate (OxE) drug from aqueous solutions. The nanoparticles were characterized by Fourier transform infrared (FTIR) spectroscopy, field emission gun scanning electron microscopy (FEG-SEM), N_2_ porosimetry, ζ-potential (ZP), thermogravimetric analysis (TGA/DTG), and X-ray diffraction (XRD) to assess their textural, structural, morphological, and thermal properties. Moreover, their *in vitro* biocompatibility was evaluated using SH-SY5Y cells. Li_2_O-NPs exhibited mesoporous morphology with small particle clusters, characteristic peaks of the antifluorite crystalline phase, a negative surface charge, and functional groups consistent with Li_2_O. Cytotoxicity assays confirmed that the nanoparticles provided a biocompatible environment, causing no oxidative stress or inflammation with the tested concentration range (1–150 μg mL^–1^). Regarding the adsorption equilibrium, analysis showed that OxE adsorption onto Li_2_O-NPs followed the Hill model (*q_H_* = 38.96 mg g^–1^, *K_H_* = 0.0384 mg L^–1^, *n*_*H*_ = 0.5582, *R* ^2^ = 0.9975), indicating moderate affinity between the adsorbate and nanoadsorbent. Kinetics studies revealed that the pseudo-first-order (PPO) model provided the best fit, with *q*_1_ = 130.7 mg g^–1^ and *k*_1_ = 0.0061 min^–1^, indicating physical adsorption as the predominant mechanism for the OxE removal. Therefore, Li_2_O-NPs demonstrated strong potential as a nanoadsorbent for the treatment of water contaminated with stable pharmaceutical compounds.

## Graphical abstract

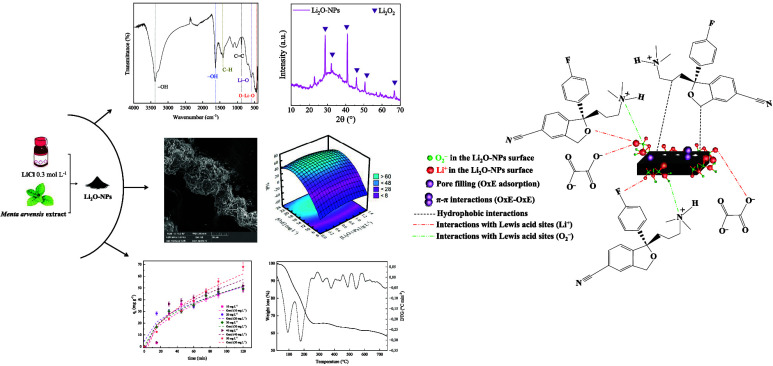

## 1 Introduction

The demand for psychiatric drugs has been steadily increasing and increasingly sought after for the treatment of mental illnesses caused by stress, anxiety, and depression.^1^ Escitalopram oxalate drug (C_20_H_21_FN_2O_, OxE) is widely prescribed for the treatment of these mental disorders, since increasing serotonin levels in the brain helps to improve mood and alleviate symptoms of anxiety and depression.^2^ However, the disposal of this drug has raised significant environmental concerns. OxE is toxic, chemically stable, poorly biodegradable, and difficult to remove through conventional wastewater treatment methods.^3^

In view of these challenges, the adsorption process has gained prominence as a promising method for pollutant removal. It offers operational simplicity, high efficiency, versatility, and the possibility of using alternative materials, such as biosorbents.^4^ Nanotechnology applied to adsorption has emerged as an advanced approach, exploiting nanomaterials to capture, store, and remove specific substances from solutions, gases, or surfaces. Furthermore, nanotechnology facilitates the functionalization of these materials, contributing to more sustainable and effective solutions in several sectors.^5^

Among the promising nanoadsorbents, lithium oxide nanoparticles (Li_2_O-NPs) stand out for their favorable chemical properties, including high basicity, chemical stability, and tunable surface reactivity, making them ideal for wastewater treatment.^6^ In this study, Li_2_O-NPs were synthesized using a green synthesis method involving the *Mentha arvensis* extract. This plant extract acts as a bioreducing agent for the metal precursor. This plant was chosen due to its high reducing potential and its rich content due to the presence of bioactive compounds such as flavonoids and terpenoids, which allow an ecological synthesis that aids in the process of nanoparticle formation.^7^

In addition to their potential in wastewater treatment, Li_2_O-NPs are also of interest in the medical field, particularly for the treatment of bipolar disorder. Lithium compounds are well known for their mood-stabilizing properties, which affect the functioning of neurotransmitters in the brain. This dual application of Li_2_O NPs, both in environmental remediation and as a therapeutic agent, highlights their versatile potential.

The main objective of this study is to synthesize and characterize a novel form of Li_2_O NPs derived from the *M. arvensis* extract for the adsorption of OxE. Furthermore, this study aims to evaluate the *in vitro* safety profile of these nanoparticles using the SH-SY5Y neuronal cell line. The novelty of this research lies in the dual approach of utilizing Li_2_O NPs for efficient wastewater treatment, contributing to achieving clean water and sanitation (Sustainable Development Goal 6), while investigating the potential neurotoxic effects of these nanomaterials. By addressing the environmental and biological implications, this study provides valuable insights into the feasibility of using Li_2_O NPs in real-world applications.

## 2 Materials and Methods

### 2.1 Preparation of the *M. arvensis* Extract

Mint leaves (*M. arvensis*) were used to make the extract by the infusion method.^8^ They were collected on a local property in Santa Maria, RS, Brazil. Thus, 20 g of mint leaves and 200 mL of distilled water were mixed under magnetic stirring at 300 rpm and 75 ± 2 °C for 20 min.

### 2.2 Biosynthesis of Li_2_O-NPs

Figure 1 shows the biosynthesis of Li_2_O-NPs from the *M. arvensis* extract, according to the literature.^9^ 200 mL of the *M. arvensis* extract and 200 mL of a lithium chloride solution (LiCl, 0.3 mol L^–1^, Sigma-Aldrich, ReagentPlus, 99%) were mixed under magnetic stirring (200 rpm, 25 ± 2 °C for 2 h). At the stabilization step, the precipitate was dried (80 °C for 72 h) and calcinated (400 °C/2 h/10 °C min^–1^).

**Figure 1:**
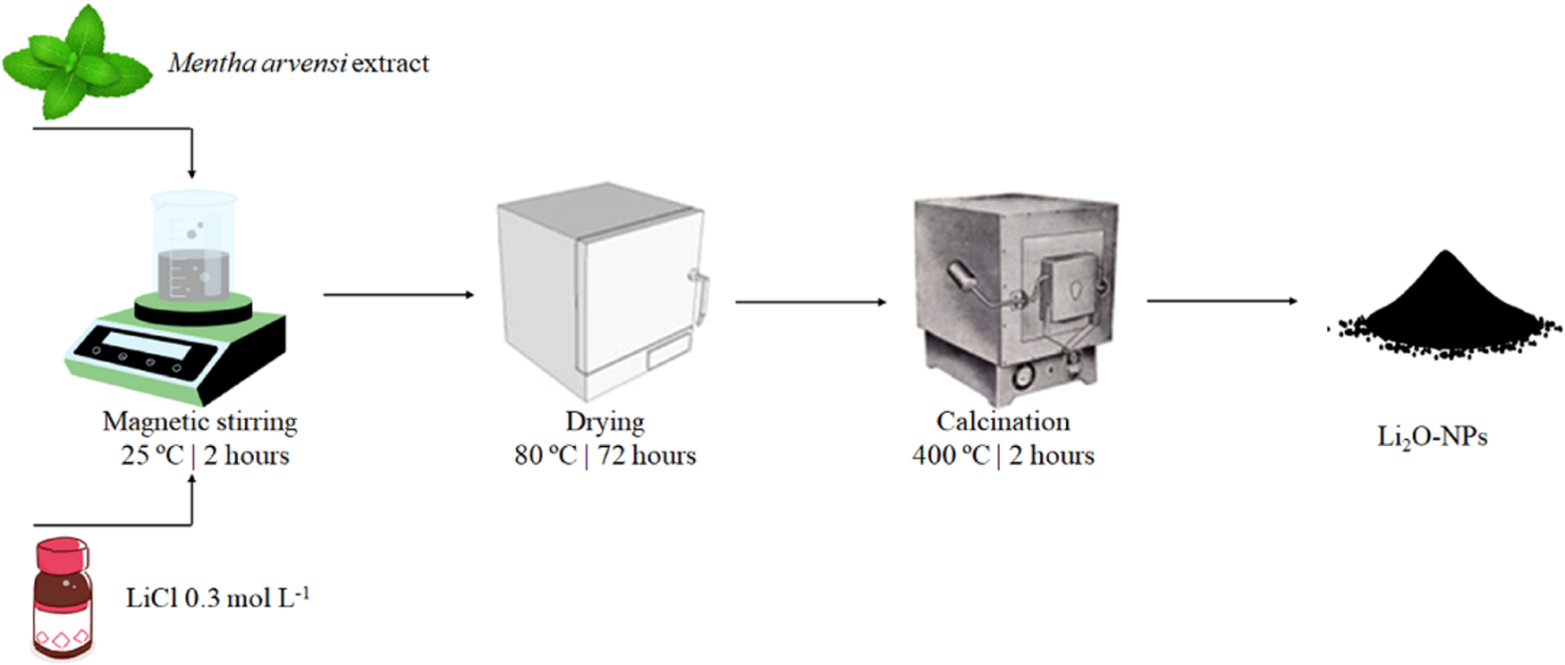
Representation of Li_2_O-NPs from the *M. arvensis* extract.

### 2.3 Characterization

Li_2_O-NPs were characterized by Fourier transform infrared (FTIR) spectroscopy, field emission gun scanning electron microscopy (FEG-SEM), N_2_ porosimetry, ζ-potential (ZP), thermogravimetric analysis (TGA/DTG), and X-ray diffraction (XRD). The methodology of characterization techniques is supplied as Supporting Information (SI).

### 2.4 Experimental Design by CCRD

To determine the ideal condition of the OxE removal by the adsorption process was carried out an experimental design by Central Composite Rotatable Design (CCRD 2^2^), where the OxE and Li_2_O-NPs concentration were used as independent variables, and the percentage of OxE removal (%R) was the response variable, according to Table 1.

**Table 1:** Coded Treatments Used in CCRD 2^2^

	[OxE] (mg L^–1^)	[Li_2_O-NPs] (g L^–1^)
–1.41	7.9	0.03
–1	10.0	0.2
0	15.0	0.6
1	20.0	1.0
+1.41	22.1	1.2

### 2.5 Adsorption Tests

OxE (CAS Number 219861–08–2, Weight-Average: 414.4 g mol^–1^, commercial tablet) was used as a target pollutant and Li_2_O-NPs as an adsorbent for the adsorption tests. The tests were carried out in batches at the natural pH of the OxE solution (pH 6.97). Aliquots were collected at times 0, 15, 30, 45, 60, 75, 90, and 120 min and filtered with ϕ = 0.45 μm. Ultraviolet–visible (UV–vis) spectroscopy was used to evaluate the OxE adsorption in a Shimadzu 1280 spectrophotometer at λ = 238 nm.^10,11^ Moreover, the percentage of OxE removal was calculated as shown in eq 1.

1 $$\%R=\frac{C_{0}-C_{t}}{C_{0}}\times 100$$

where *C*_0_ is the initial OxE concentration (mg L^–1^) and *C*_t_ is the OxE concentration (mg L^–1^).

### 2.6 Kinetic and Equilibrium Adsorption

The adsorption equilibrium and kinetics data were analyzed using the models presented in the Supporting Information (SI) and the kinetic and equilibrium parameters were determined by fitting the models with the experimental data using nonlinear regression. The calculations were performed using Statistica 10 software (StatSoft). Coefficient of determination (*R* ^2^), adjusted coefficient of determination (*R*_adj_^2^), average relative error (ARE), and the sum of square error (SSE) were used to evaluate the model’s fit quality.

### 2.7 In Vitro Safety Profile and Statistical Analysis

The methodology of biological tests with neuron-like cells (SH-SY5Y) and statistical analysis is supplied as Supporting Information (SI). Briefly, *in vitro* safety profile tests were performed on Li_2_O-NPs, such as cell viability, reactive oxygen species (ROS) generation, and NO generation and determination of dsDNA extracellular release.

## 3 Results and Discussion

### 3.1 Characterization of Li_2_O-NPs

Figure 2a–c shows the XRD diffractogram, FTIR spectrum, and FEG-SEM micrography.

**Figure 2:**
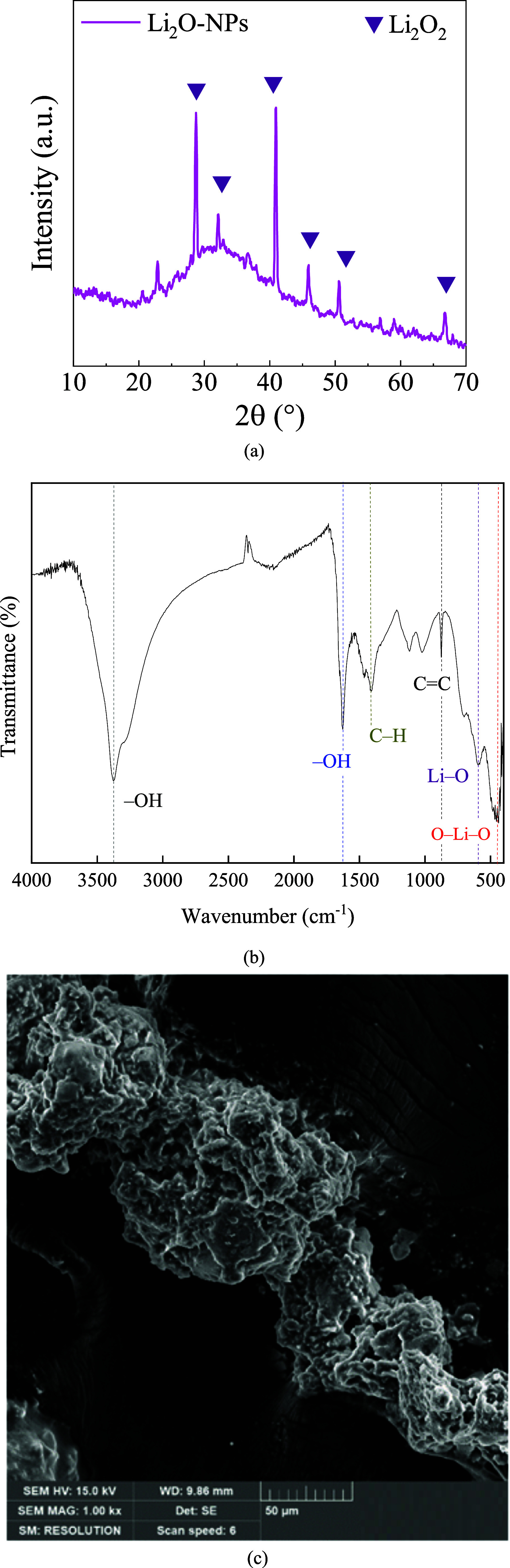
(a) XRD diffractogram, (b) FTIR spectrum, and (c) FEG-SEM micrography of Li_2_O-NPs from the *M. arvensis* extract.

According to Figure 2a, the XRD diffractogram identified the antifluorite phase (Li_2_O_2_, JCPDS 01–073–1640) at 2θ° = 28.12° (101), 32° (101), 42° (102), 46° (200), 44° (112), 53° (004), and 68° (114). The lattice parameter was calculated from the peak at 2θ = 28.12° (101) using the Bragg equation obtaining *a* = 4.48 Å, and the average crystallite size was estimated by the Scherrer equation, resulting in 5.85 ± 1.2 nm.^12^ The FTIR spectrum in Figure 2b shows the following functional groups of LiO_2_–NPs, where (i) the stretching vibration of hydroxyl (^•^OH) of water adsorbed onto Li_2_O-NPs surface (LiOH) at 3400 and 1600 cm^–1^; (ii) C–H asymmetrical stretching at 1500–1400 cm^–1^ of alkane; (iii) C=C stretching vibration of the alkene at 900 cm^–1^; (iv) Li–O symmetrical stretching at 700–800 cm^–1^, and O–Li–O (symmetrical bending at 500–600 cm^–1^) from the metallic precursor. Li_2_O-NPs showed an irregular surface and the presence of small clusters (Figure 2c), which is typical of metallic oxide nanoparticles.^13,14^ Table 2 presents the textural (surface area and porosity) and structural properties of Li_2_O-NPs.

**Table 2:** Textural and Surface Properties of Li_2_O-NPs from the *M. arvensis* Extract

*S*_BET_ (m^2^ g^–1^)	*D*_p_ (nm)	*V*_p_ (cm^3^ g^–1^)	*Z*_P_ (mV)
3.7 ± 0.1	41.6 ± 2.6	0.02 ± 0.002	–22.3 ± 1.4

According to Table 2, Li_2_O-NPs showed mesoporosity,^15^ and the surface area, even if not extremely high, can still be sufficient for adsorption. The pore volume indicates that there is available space within the material to store the adsorbed molecules. Regarding the ζ-potential, this indicates a moderate negative surface charge, suggesting that the particles have good stability in an aqueous medium.^16^

Figure 3 shows the DTA curve and TGA thermogram of Li_2_O-NPs, where the weight loss (around 26%) in the first temperature (100–200 °C) was probably due to the water loss from the sample and some organic residues present in the composition of Li_2_O-NPs. Moreover, about 30% weight loss up to 210 °C, remaining essentially stable from 300 to 750 °C. Furthermore, some reports in the literature describe similar thermal behavior and weight loss, in which zinc oxide (ZnO-NPs) and silver (AgNPs) nanoparticles synthesized by green methods showed initial weight reduction due to the elimination of water and organic residues up to 200 °C, followed by significant thermal stability at higher temperatures.^17^ These results suggest that green metallic nanoparticles have good thermal resistance, probably due to the stabilization caused by organic compounds present in the composition of the plant extracts.

**Figure 3:**
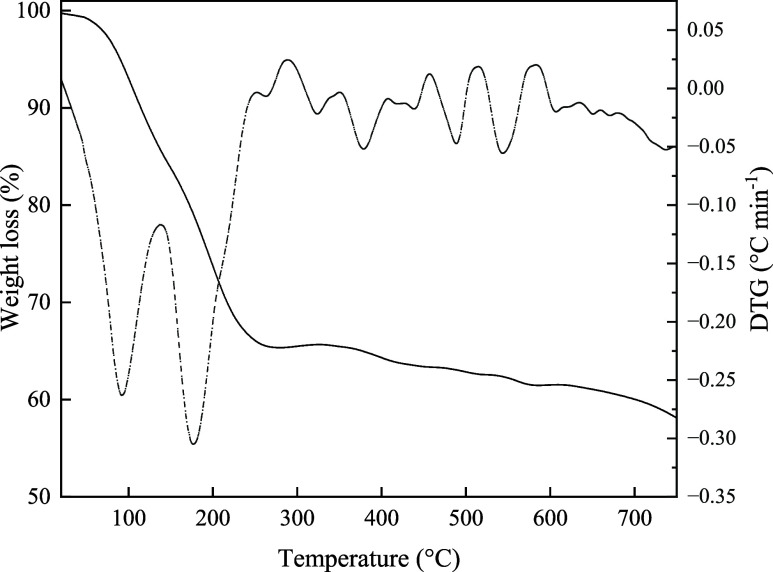
DTA curve and TGA thermogram of Li_2_O-NPs.

### 3.2 CCRD 2^2^ and OxE Adsorption

Table 3 presents the CCDR 2^2^ for the OxE adsorption, where adsorption capacity (*q*_e_) was used as a response variable.

**Table 3:** CCRD 2^2^ for OxE Adsorption

[OxE] (mg L^–1^)	[Li_2_O-NPs] (g L^–1^)	%*R*
20.00	0.20	49.06
15.00	0.60	44.33
15.00	0.30	53.52
15.00	0.60	71.01
22.07	0.60	62.69
10.00	1.00	60.43
15.00	1.17	67.70
20.00	1.00	46.39
10.00	0.20	96.88
7.93	0.60	20.51
15.00	0.60	48.89

According to Table 3, the ideal conditions were [OxE] = 10 mg L^–1^ and [Li_2_O-NPs] = 0.20 g L^–1^ achieving 96.88% removal. Figure 4a,b shows the Pareto chart and three-dimensional (3D) surface response for the OxE adsorption by CCRD 2^2^.

**Figure 4:**
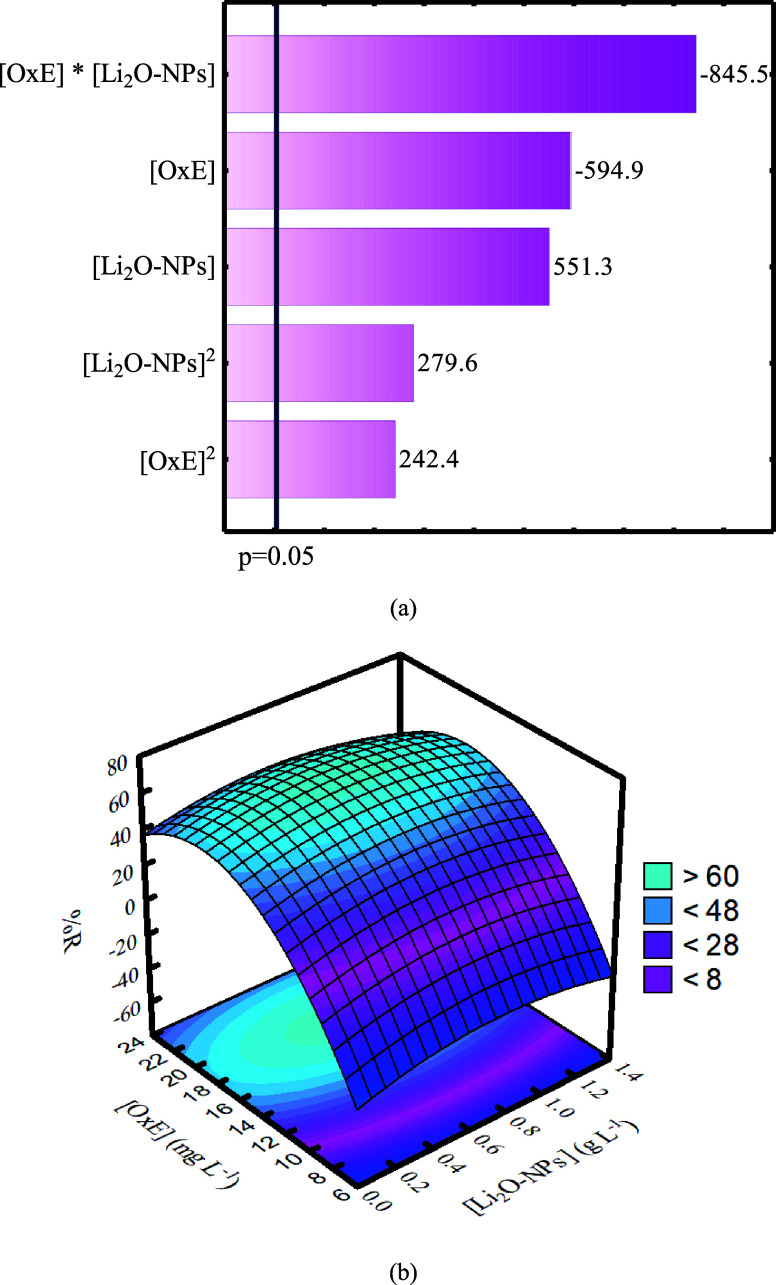
(a) Pareto chart and (b) 3D surface response by CCRD with 2^2^.

According to the Pareto chart (Figure 4a), all variables were significant to the adsorption process. In this view, [OxE] and the interaction between [OxE] × [Li_2_O-NPs] showed a negative and indirect relationship with the response. Thus, as the [OxE] values increase and the [Li_2_O-NPs] values remain constant, the number of molecules in the solution increases, causing an imbalance between the number of adsorbate molecules and the available active sites on the nanoadsorbent.^18^

In parallel, these results suggest that a simultaneous increase in these variables should reduce the OxE removal, probably due to the agglomeration of nanoadsorbent particles or supersaturation of the adsorbent surface at higher [OxE] levels, reducing the active sites available for effective adsorption.^19^ On the contrary, [Li_2_O-NPs] showed a positive and direct influence on the OxE removal, suggesting that an increase in [Li_2_O-NPs] might increase the OxE removal by adsorption. It is due to the increase of the number of available active sites on the nanoadsorbent, especially for a narrow range of nanoadsorbent concentrations (*e.g*., adsorbent concentration range used in this work: 0–1.17 g L^–1^).^20^

Furthermore, [Li_2_O-NPs]^2^ and [OxE]^2^ have a positive and direct effect on the removal, indicating that an abrupt increase in both variables can increase the values for the adsorption capacity and, hence, the drug removal. This result is extremely associated with the nanoadsorbent concentration range used in the experimental run, which was quite narrow (*e.g*., from 0.2 to 1.0 g L^–1^, with slight extrapolations), in which the increase in [Li_2_O-NPs] will result in a higher number of available active sites on the solid without reaching oversaturation of the solid surface or significant particle agglomeration.^21^ However, for extremely high values of [Li_2_O-NPs], such as 1.5–3 g L^–1^, these phenomena probably might be expected.^22^

According to Figure 4b, it can be observed that the adsorbate and nanoadsorbent concentrations had a great influence on the removal of the pollutant, in which higher values for drug removal (%R) are achieved for higher [OxE] values (*e.g*., [OxE] = 16–20 mg L^–1^). This tendency occurs in response to an increase in the gradient concentration of OxE in solution, which increases the diffusion rate, thus facilitating the transfer of the adsorbate molecules from the solution to the surface of the Li_2_O-NPs nanoadsorbent.^23^ However, for all nanoadsorbent concentration ranges presented in the surface response, around 40–60% drug removal is achieved, since [OxE] lies on 16–20 mg L^–1^. This suggests that the LiO_2_–NPs nanoadsorbent shows a great number of active sites.^24^

### 3.3 Kinetic Adsorption

Figure 5a–c shows the kinetic curve for the OxE adsorption onto Li_2_O-NPs. Meanwhile, the estimated kinetic parameters are shown in Table 4.

**Figure 5:**
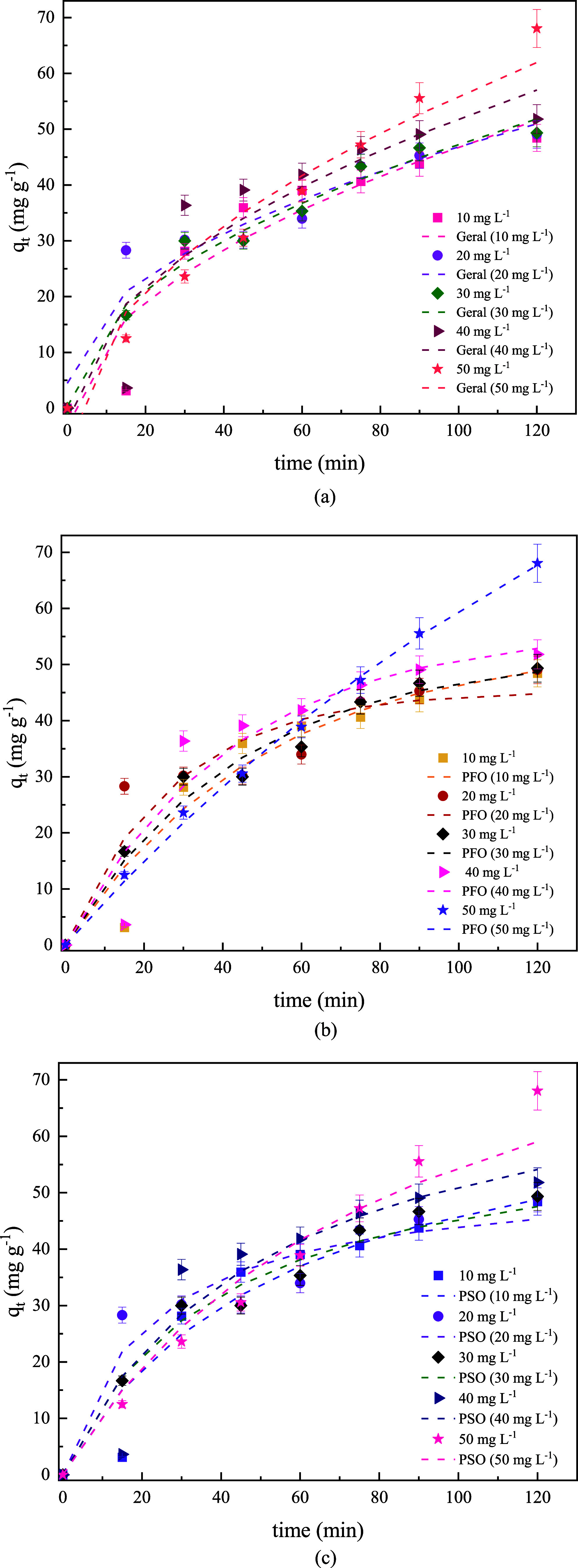
Kinetic curves for OxE onto Li_2_O-NPs for (a) Geral, (b) PFO, and (c) PSO models (*T* = 25 °C; [Li_2_O-NPs] = 0.20 g L^–1^, and pH 6.97).

**Table 4:** Kinetic Parameters for the Adsorption of OxE on Li_2_O-NPs

kinetic models	initial OxE concentration (mg L^–1^)				
PFO model	10	20	30	40	50
*q*_1_ (mg g^–1^)	53.761	45.355	51.997	56.307	130.71
*k*_1_ (min^–1^)	0.0200	0.0363	0.0228	0.0233	0.0060
*R* ^2^	0.9981	0.8863	0.9758	0.9160	0.9981
*R*_adj_^2^	0.9973	0.8408	0.9661	0.8824	0.9973
SSE	6.957	186.82	46.412	240.88	6.957
ARE (%)	0.8697	10.873	6.2811	48.739	3.047
PSO model	10	20	30	40	50
*q*_2_ (mg g^–1^)	71.817	53.535	63.090	77.346	101.703
*k*_2_ (g mg^–1^ min^–1^)	0.00024	0.00087	0.00040	0.00025	0.00011
*R* ^2^	0.9913	0.9222	0.9772	0.9066	0.9627
*R*_adj_^2^	0.9891	0.8911	0.9681	0.8693	0.9477
SSE	132.9	127.83	43.687	267.73	132.86
ARE (%)	16.61	9.7030	5.9645	51.907	8.947
general kinetic model	10	20	30	40	50
*K*_*D*_ (mg g^–1^ min^–0.5^)	5.00823	4.24586	4.70030	5.41273	6.31608
*C* (mg g^–1^)	–3.2378	4.6822	0.33244	–2.2729	–7.2212
*R* ^2^	0.9542	0.9340	0.9797	0.8699	0.9542
*R*_adj_^2^	0.9359	0.9076	0.9716	0.8178	0.9359
SSE	163.08	108.43	38.946	373.00	163.07
ARE (%)	20.38	7.9362	6.0707	58.835	11.344

According to Figure 5 and Table 4, based on the analysis of the statistical parameters (*R* ^2^ = 0.9981 | *R*_adj_^2^ = 0.9973) and the smallest errors (SSE = 6.9576 | ARE = 0.8697%) the pseudo-first-order (PPO) model was the best fit for adsorption data, which is highlighted by the largest coefficients of determination. Thus, the theoretical adsorption capacity and kinetic rate constantly evaluated from the nonlinear regression were *q*_1_ = 130.7 mg g^–1^ and *k*_1_ = 0.0061 min^–1^, respectively. In the model, *q*_e_ increases from 71.817 mg g^–1^ (10 mg L^–1^) to 130.71 mg g^–1^ (50 mg L^–1^). This is because as the concentration increases, there are more OxE molecules available in the solution to interact with the active sites of the adsorbent. Furthermore, this result indicates that the adsorption rate of OxE is proportional to the difference between the adsorbed amount and the maximum adsorption capacity, with the process occurring at a constant rate and governed by the rate constant.^25^ Furthermore, the theoretical and experimental *q*_e_ values showed a physical adsorption mechanism, probably due to diffusion interactions and in the order of magnitude of van der Waals forces.^26^

### 3.4 Equilibrium Adsorption

Equilibrium of adsorption was carried out to comprehend the mechanism involved in the OxE adsorption and evaluate the properties of the nanoadsorbent. Table 5 shows the parameters evaluated from the nonlinear regression of data of adsorption for different drug concentrations.

**Table 5:** Equilibrium Parameters for the Adsorption of OxE on Li_2_O-NPs^a^

langmuir model		freundlich model	
*q*_max_ (mg g^–1^)	51.52	*K*_*F*_ (mg g^–1^) (mg L^–1^)^1/*n*^	38.88
*K*_*L*_ (L mg^–1^)	3.017	*1/n*	11.59
*R* ^2^	0.6690	*R* ^2^	0.9049
*R*_adj_^2^	0.3380	*R*_adj_^2^	0.8098
SSE	0.2429	SSE	0.1469
ARE (%)	4.8575	ARE (%)	2.9473
hill model		liu model	
*q*_*H*_ (mg g^–1^)	38.96	*qm*_*L*_ (mg g^–1^)	31.472
*K*_*H*_ (mg L^–1^)	0.0384	*K*_*g*_ (L mg^–1^)	8.0680
*n*_*H*_	0.5582	n_L_	0.0862
*R* ^2^	0.9975	*R* ^2^	0.9041
*R*_adj_^2^	0.9949	*R*_adj_^2^	0.8082
SSE	0.3394	SSE	12.728
ARE (%)	0.0678	ARE (%)	2.5456
Sips Model			
	*q*_*max*_ (mg g^–1^)	31.47	
	*K*_*s*_ (L mg^–1^)	8.068	
	*m*	0.0862	
	*R* ^2^	0.9049	
	*R*_adj_^2^	0.8098	
	SSE	12.729	
	ARE (%)	2.5457	

a [Li_2_O-NPs] = 0.20 g L^–1^ | [OxE] = 10–50 mg L^–1^ | 298 K | pH 6.97.

According to Table 5, the Hill model was the best fit for experimental data, which is confirmed by the high determination coefficient values (*R* ^2^ = 0.9975 and *R*_adj_^2^ = 0.9949). Moreover, the Hill isotherm showed the smallest values for the metrics SSE (0.3394) and ARE (0.0678%). Thus, the maximum adsorption capacity of Li_2_O-NPs for OxE drug was *q*_H_ = 38.96 mg g^–1^, which is comparable to the maximum adsorption capacity of other green metallic nanoadsorbents and nanocomposites used for drug removal (40 mg g^–1^).^27^ Moreover, the Hill constant (*K*_*H*_) indicates the adsorbent’s affinity for the adsorbate.^28^ Thus, a positive value (*K*_*H*_ = 0.0384 mg L^–1^) suggests favorable adsorption at the studied equilibrium concentrations.

In parallel, the cooperativity coefficient (*n*_*H*_) reflects a negative interaction or a lack of cooperation between adsorbed molecules, in which values less than indicate negative interaction or noncooperation among adsorbed molecules, meaning site occupancy reduces the likelihood of further adsorption.^29^ In this study, *n*_*H*_ = 0.5582, indicating that the site occupancy reduces the likelihood of additional adsorption, highlighting a moderate affinity between adsorbate and nanoadsorbent and reduced cooperative behavior during the adsorption process.

According to Figure 6, the adsorption of OxE involves physical interactions (hydrophobic and van der Waals forces) with escitalopram and the oxalate group, π–π interactions between aromatic rings of escitalopram molecules, and interaction between OxE and Lewis acid sites attributed to surface Li and O atoms (*e.g*., Li^+^ and O_2_^–^). Additionally, pore filling plays a key role in the drug adsorption mechanism.

**Figure 6:**
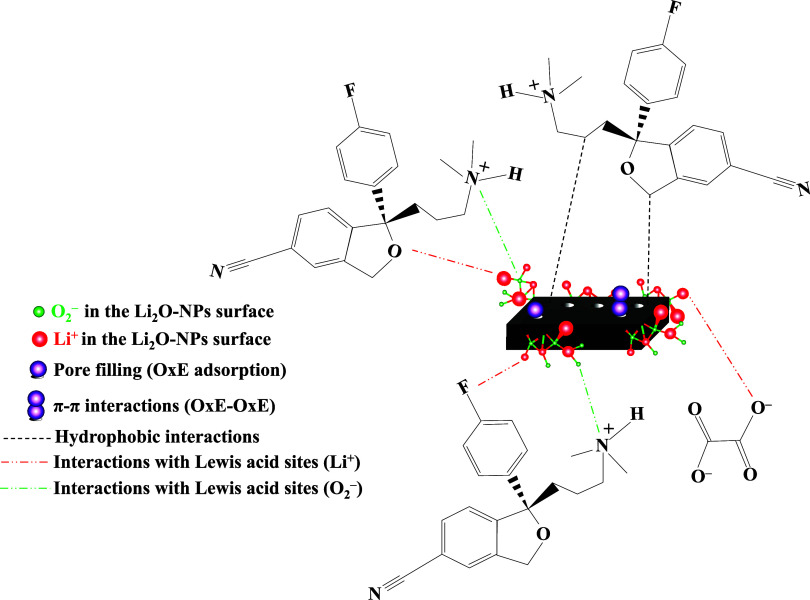
Proposed mechanism for OxE adsorption.

Table 6 presents a comparative study between escitalopram and its analogues, highlighting the isotherms used and other significant parameters.

**Table 6:** Comparative Studies on Drug Adsorption

adsorbent	drug	mechanisms	comments	refs
magnetic biochar from waste derived from *Aloe vera* leaves	escitalopram oxalate (OxE), paroxetine, and fluoxetine	Pseudo-second-order, chemisorption mechanism	*q*_máx_ (mg g ^–1^) escitalopram 37.1, paroxetine 31.5, fluoxetine 34.1	^30^
green functionalized carbon nanotubes (CNTs) from coffee extract	losartan (LOS) and diclofenac (DIC)	Pseudo-second-order, chemisorption mechanisms	*q*_max_ 76.99 mg g^–1^ (DIC) and 92.19 mg g^–1^ (LOS)	^31^
Co–Zn–Ni trimetallic oxide nanoparticles using *Cicer arietinum* leaf extract	ciprofloxacin	antibiofilm activity	the antibiofilm activity was 51.6% and the antibiotic ciprofloxacin 42.5%	^32^
layered double hydroxide (LDH)	delafloxacin (DLX)	langmuir–Freundlich	*q*_max_ 957.82 mg g^–1^	^33^
CuCoFe-MOF/CoFe_2_O_4_/banana peel activated carbon (BPAC) composite-modified carbon paste electrode (CPE)	ribociclib	differential pulse voltammetry (DPV) showed a wide linear range of 0.2–9.7 μmol L^–1^ and a limit of detection (LOD) of 0.025 μmol L^–1^	achieving recoveries between 98.6% and 101.8% with an RSD below 2.0%.	^34^
green multiwalled carbon nanotubes	ciprofloxacin (CIP) and ofloxacin (OFL)	sips and langmuir–freundlich fit the data better	single-component systems: 0.433 mmol CIP g^–1^ and 0.457 mmol OFL g^–1^; multicomponent system: 0.958 mmol CIP g^–1^ and 0.872 mmol OFL g^–1^	^35^
lithium oxide (Li_2_O-NPs) from the *M. arvensis* extract	escitalopram oxalate	physical adsorption mechanism	96%/38.96 mg g^–1^	this study

Table 6 presents a variety of adsorbents, including functionalized carbon nanotubes, layered double hydroxides (LDH), metal oxides, magnetic biochar, and MOF-based composites. The main study is different because it uses nanostructured lithium oxide (Li_2_O-NPs) obtained from the *M. arvensis* extract, a plant-based material, which suggests a sustainable potential. The adsorbed drugs include antibiotics such as ciprofloxacin, ofloxacin, and delafloxacin, antihypertensives such as losartan and diclofenac, and ribociclib, a CDK4/6 inhibitor.

The adsorption mechanisms vary between chemisorption, electrostatic interactions, and physical adsorption, with some studies modeling the data using Langmuir–Freundlich isotherms and kinetic models such as pseudo-second-order. Comparing the results presented in the table with those of the main study, we can observe some differences and similarities in terms of adsorption efficiency, adsorptive capacity, and mechanisms involved.

The main study reports an adsorption efficiency of 96% and an adsorption capacity of 38.96 mg g^–1^ for escitalopram oxalate on nanostructured lithium oxide (Li_2_O-NPs). Compared with other systems in the table, these values are competitive, although they are lower than some specific adsorbents. Thus, the main study presents a competitive adsorption efficiency (96%), but a lower adsorptive capacity (38.96 mg g^–1^) when compared with other materials that involve stronger chemical interactions. The main distinction lies in the physical adsorption mechanism, which may be advantageous for adsorbent reuse and regeneration processes, making it potentially more sustainable and economically viable in certain applications.

### 3.5 In Vitro Safety Profile

MTT in 24 h (Figure 7a) had an increase in viability at 50, 100, and 150 μg mL^–1^ in relation to the negative control; in 48 h (Figure 7b), there was a decrease in proliferation at 50 and 150 μg mL^–1^ in relation to the negative control; in 72 h (Figure 7c), there was a decrease in proliferation in practically all concentrations except in 150 μg mL^–1^. These results suggest that Li_2_O-NPs may affect cell viability and proliferation in a dose-dependent manner. The toxicity of nanomaterials is not only related to their size but also related to other factors such as their morphology, concentration, and exposure time.^36^

**Figure 7:**
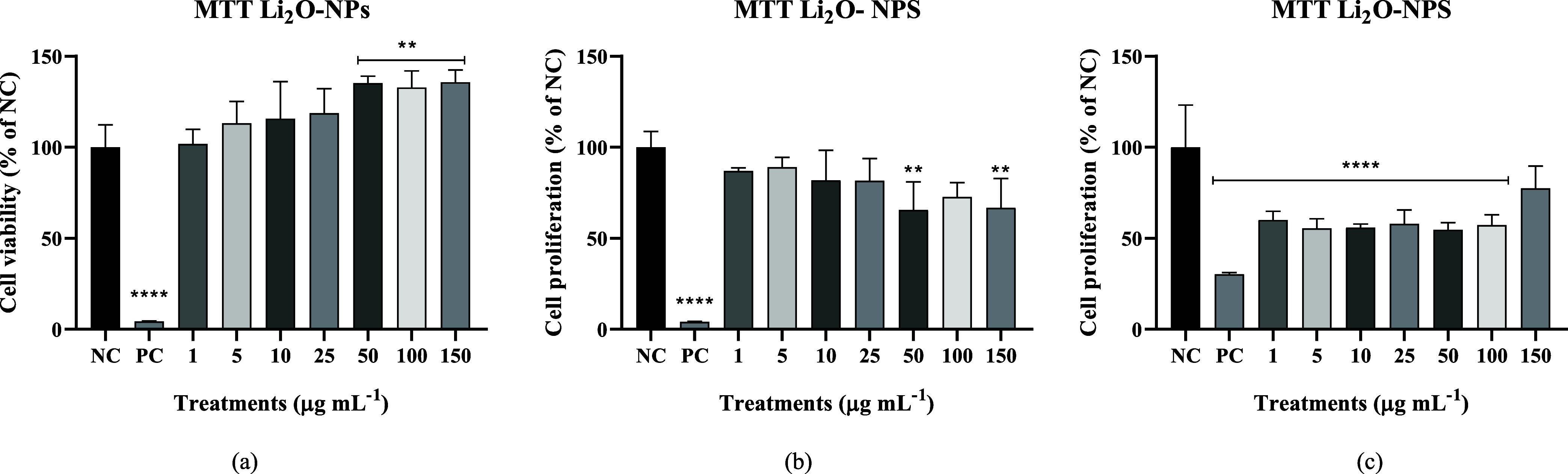
Cell viability and proliferation via MTT assay: (a) Results at 24 h, (b) 48 h, and (c) 72 h. NC: only culture medium and PC: hydrogen peroxide (3 mol L^–1^).

These results suggest that Li_2_O-NPs may affect cell viability and proliferation in a dose-dependent manner and mainly time-dependent, which is found in other studies evaluating the cytotoxicity of nanomaterials.^37^

The MTT assay is an important viability analysis experiment. It is the most widely used tetrazolite salt for determining cell safety.^38^ This viability analysis is essential for assessing the safety of nanomaterials, since the MTT test is recommended by NanoReg.^39^ Thus, metal nanoparticles are commonly evaluated for their toxicity using the MTT assay.^40^

Although the MTT assay provides important information on cell viability, it is important to carry out other assays that can then determine by which route the cells are being killed, for example, for analyzing oxidative metabolism.

For the DCF results in 24 h (Figure 8a), we obtained an increase in the levels of reactive oxygen species in 5 μg mL^–1^ in relation to the negative control; at the other times, all concentrations were equal to the negative control. These results suggest that Li_2_O-NPs did not promote substantial ROS generation in cells, which may be advantageous for biomedical applications since low oxidative stress indicates lower oxidative stress-related cytotoxicity. In 5 μg mL^–1^, there was a small increase in ROS generation, but the effect is reduced at higher concentrations, which is interesting for the safe use of these nanoparticles in biological environments. At 48 and 72 h (Figure 8b,c), no changes in concentrations were observed. Oxidative stress is related to several problematic factors in cells, such as increased inflammation, since these are feedback events. If there is no production of ROS, this is a positive factor for the sample in question, as it is directed toward cell safety, should these NPS meet the cells.^41^

**Figure 8:**
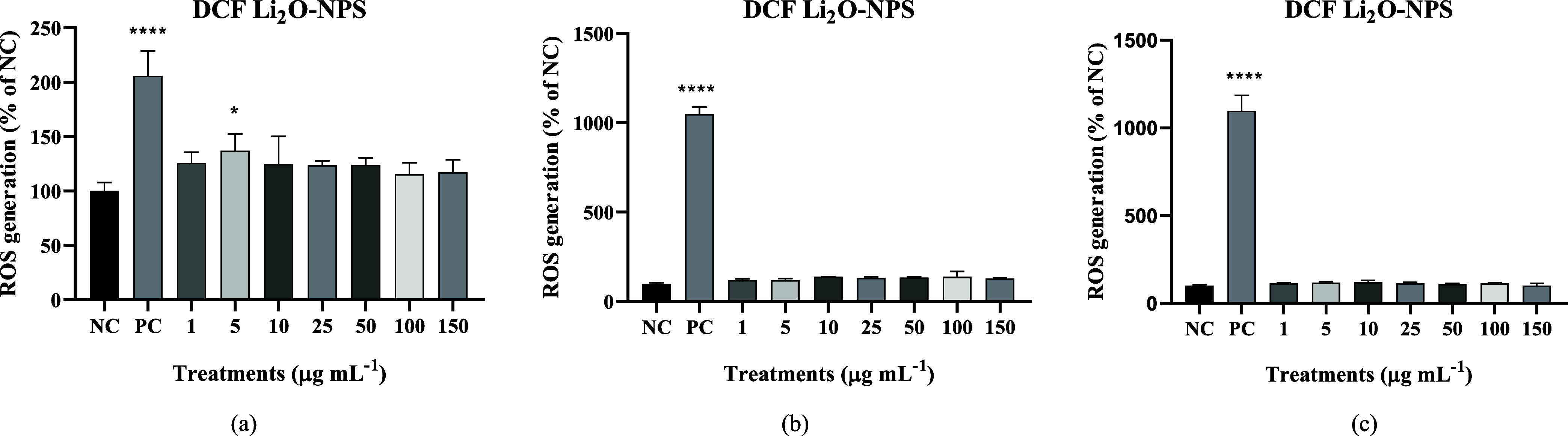
ROS levels via DCF assay: (a) Results at 24, (b) 48 h, and (c) 72 h. NC: only culture medium and PC: hydrogen peroxide (3 mol L^–1^).

In the nitric oxide test (Figure 9), the only concentration that increased levels was 150 μg mL^–1^ after 48 h (Figure 9b), and the other concentrations remained at basal levels, meaning they were the same as the negative control. Among the cellular signals that the nitric oxide molecule is involved, the indication of inflammation and oxidative metabolism are two of them, so these results indicate that Li_2_O-NPs did not induce a significant inflammatory response in cells^42^ because it remains low at all Li_2_O-NPs concentrations tested. Only the positive control showed a notable increase in NO, which validates the assay. This suggests that Li_2_O-NPs may be relatively safe for use in biological applications, since they do not promote significant inflammatory stress.

**Figure 9:**
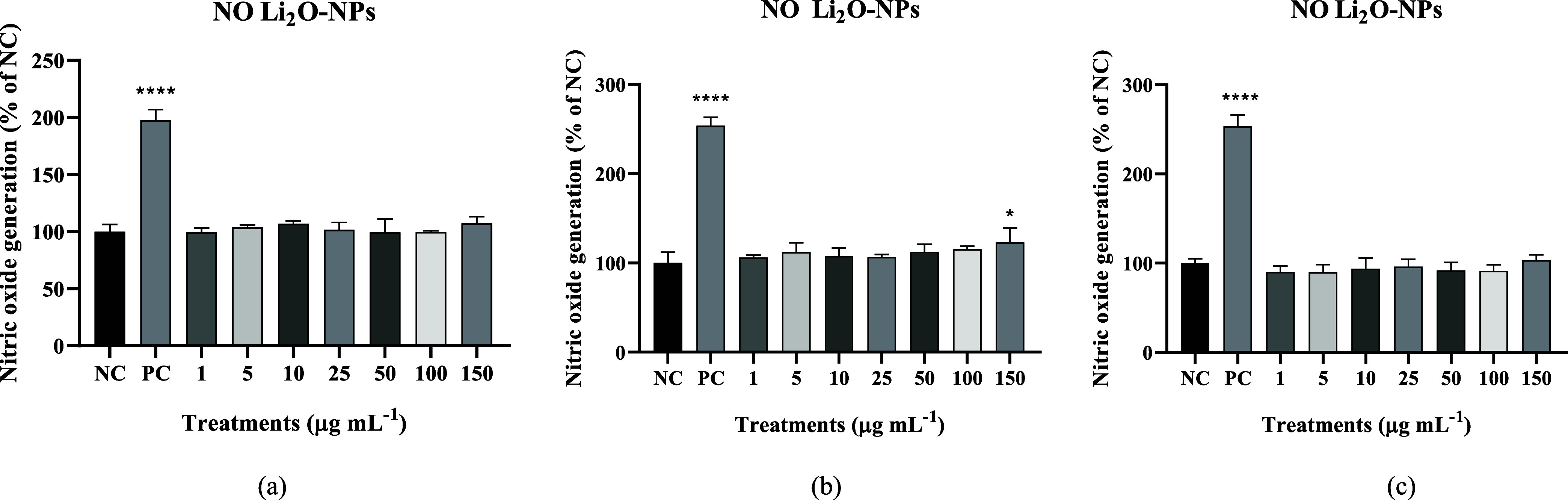
Nitric oxide levels via NO assay: (a) Results at 24 h, (b) 48 h, and (c) 72 h. NC: only culture medium and PC: sodium nitroprusside (10 mg mL^–1^).

The results of the extracellular dsDNA assay in 24 h (Figure 10a) show that no concentration was able to increase the level of extracellular dsDNA release (double-stranded DNA). In 48 h (Figure 10b), we obtained a decrease in dsDNA in all concentrations except in 150 μg mL^–1^, meaning that there was a smaller cell rupture than in the negative control, and in 72 h (Figure 10c), concentrations except 25 μg mL^–1^ decreased dsDNA levels in the extracellular medium. The effect appears to be inhibitory at higher concentrations, with statistical significance being indicated. The analysis of the percentage of dsDNA in the extracellular medium suggests the rupture of the cells, as this exposes their genetic material.^43^ Thus, the effect presented at 48 and 72 h shows that the amount of dsDNA in the medium is even lower than that of the negative control, so these concentrations are beneficial for the cells.

**Figure 10:**
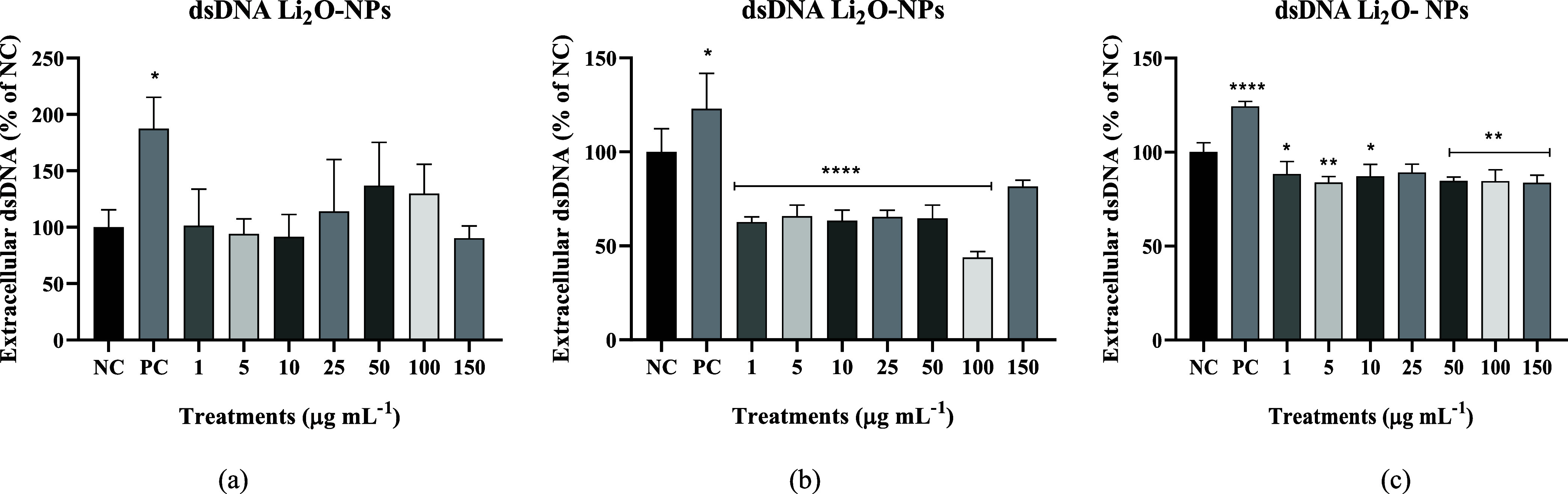
Extracellular dsDNA levels by PicoGreen assay: (a) Results at 24 h, (b) 48 h, and (c) 72 h. BNC n, where NC: only culture medium; PC: hydrogen peroxide (3 mol L^–1^).

Thus, since there was no increase in ROS, NO, and dsDNA, it could be indicated that the morphology (see Figure 2c) of the nanoparticles may have influenced their cytotoxicity in the 72 h MTT test because although there are no spikes on the surface, which could substantially impair cell viability, there are small clusters, which in higher concentrations and for longer exposure to cells may have prevented their normal growth.^44^

## 4 Conclusions

According to the results of the adsorption kinetics and equilibrium, it can be inferred that the adsorption of OxE occurs due to physical mechanisms, with the maximum adsorption capacity being 39.48 mg g^–1^, as reported by the Hill model. Additionally, this study suggests that the adsorbate has a moderate affinity for the nanoadsorbent. In the batch adsorption test, 96% of OxE removal was reported under the ideal conditions of CCRD 2^2^ ([OxE] = 10 mg L^–1^, [Li_2_O-NPs] = 0.2 g L^–1^, pH 6.97, *T* = 298.15 K). In parallel, the SHSY-5Y neural cells showed an increase in cell viability, causing practically no significant damage, confirming that the nanoparticles provided a favorable environment. Among these, green lithium oxide nanoparticles (Li_2_O-NPs) are promising for use in alternative treatments for removing organic pollutants, such as pharmaceuticals, in wastewater in addition to being safe for industrial application.

## Supplementary Material



## Data Availability

The data that support the findings of this study are available on request from the corresponding author.

## Notes

A novel green lithium oxide nanoparticles for adsorption of the escitalopram oxalate and in vitrosafety profile; kinetic and equilibrium adsorption; cell culture and treatments; cellular viability determination (PDF)
