# Supplementary material for: A Novel Green Lithium Oxide Nanoparticle for Adsorption of the Escitalopram Oxalate and In Vitro Safety Profile
Source: ACS Omega. 2025 Sep 9;10(37):42335–45. doi: 10.1021/acsomega.5c02918 (PMC12461387; doi:10.1021/acsomega.5c02918)
Supplement: Supplementary file 1 [file ao5c02918_si_001.pdf]

Supporting Information (SI)

A novel green lithium oxide nanoparticles for adsorption of the escitalopram oxalate and *in vitro* safety profile

Sthéfany Nunes Loureiro[1], Leandro Rodrigues Oviedo [1], Daniel Moro Druzian[1], Yolice Patrícia Moreno[2], Giovani Pavoski[3], Denise Crocce Romano Espinosa[3], Gabriela Geraldo Sangoi[4]; Alencar Kolinski Machado[4]; William Leonardo Da Silva[1]

[1]Applied Nanomaterials Research Group (GPNAp)

Franciscan University (UFN), Santa Maria, RS, Brazil

[2]Department of Fundamental Chemistry (DQF)

Federal University of Pernambuco (UFPE), Recife, PE, Brazil

[3]Polytechnical School of Chemical Engineering

University of the Sao Paulo (USP), São Paulo, SP, Brazil.

[4]Laboratory of Cell Culture and Bioactive Effects

Franciscan University (UFN), Santa Maria, RS, Brazil

Materials and methods

Characterization

A Bruker diffractometer (model D2 Advance) with a copper tube ($\lambda_{Cu\text{-}\alpha}$ = 0.15418 nm) was used to identify the crystalline phases by X-ray diffraction (XRD) analysis ranging from 5 to 70º with an accelerating voltage of 30 kV and an applied current of 30 mA. The crystal structure was analyzed using the JCPDSCOD (International Centre for Diffraction Data) database.

Fourier-transform infrared spectroscopy (FTIR) was used to identify the functional groups in a PerkinElmer Frontier FTIR Spectrometer (transmittance mode of 4000 and 400 cm^{-1}) with 45 scans and a resolution of 2 cm^{-1}. The morphological property

was determined by Field Emission Gun – Scanning Electron Microscope (FEG-SEM) in a TESCAN MIRA3 Field Emission Scanning Electron Microscope, with magnification of 1.00 kx. To determine the textural properties (surface area, pore diameter and pore volume), N_2 porosimetry was used with relative pressure in the range of $P P_0^{-1} = 0$ to 1 measured by the Brunauer-Emmett-Teller (BET) in the Micromeritics ASAP 2020 Plus instrument. Zeta potential (ZP) was determined to use the Malvern-Zetasizer nanos model (ZEN3600, UK). The thermal properties were investigated by thermogravimetric analysis (TGA) and derivative thermogravimetry (DTG) curves on a thermogravimetric analyzer (Shimadzu, model TGA-60/60H) using a platinum sample port and synthetic air with a gas flow of 100 mL min^{-1}, over a temperature range of 0 - 750º C.

Kinetic and equilibrium adsorption

The kinetic study of adsorption was carried out by pseudo first-order (PFO) (Eq. S1) and pseudo second-order (PSO) model (Eq. S2) and general kinetic model (Eq. S3). Regarding equilibrium study, the experimental data were fitted to Langmuir (Eq. S4), Freundlich (Eq. S5), Hill (Eq. S6), Liu (Eq. S7) and Sips (Eq. S8) isotherms.

$$q_t = q_1(1 - e^{-k_1 t}) \qquad \text{(S1)}$$

$$q_t = \frac{q_2^2 k_2 t}{1 + q_e k_2 t} \qquad \text{(S2)}$$

$$q_t = K_d * t^{\frac{1}{2}} + C \qquad \text{(S3)}$$

$$q_e = \frac{q_{max} K_L C_e}{1 + K_L C_e} \qquad \text{(S4)}$$

$$q_e = K_F (C_e)^{\frac{1}{n}} \qquad \text{(S5)}$$

$$q_e = \frac{q_H * {C_e}^{n_H}}{K_H + {C_e}^{n_H}} \qquad \text{(S6)}$$

$$q_e = \frac{q_{mL}(k_g C_e)^{n_L}}{1 + (k_g C_e)^{n_L}} \quad \text{(S7)}$$

$$q_e = \frac{q_m (K_s C_e)^m}{1 + (K_s C_e)^m} \quad \text{(S8)}$$

Where: q_e is the amount adsorbed at equilibrium (mg g^{-1}); C_e is the equilibrium concentration (mg L^{-1}); q_{max} and q_{mL} are the maximum adsorption capacity of the nanoadsorbent (mg g^{-1}); k_L and k_g is the Langmuir and Liu constants (L mg^{-1}); K_F and K_s are the Freundlich and Sipes constants ((mg g^{-1}) (mg L^{-1})$^{1/n}$); K_H is the Hill adsorption constant (mg L^{-1}); n_F, n_H, n_L are the parameters associated with the adsorption strength (heterogeneity); k_1 (min^{-1}), k_2 (g mg^{-1} min^{-1}), and k_d (min^{-1})$^{1/2}$ are the first, second and *n-th* order rate constants, and q_t (mg g^{-1}) is the amount of dye adsorbed per gram of the adsorbent in time; C is a parameter related to the effective concentration of available adsorption sites on the surface of the adsorbent material

The metrics of each adjustment were determined by the coefficient of determination (Eq. S9), the adjusted determination coefficient (Eq. S10), the sum of squared error (Eq. S11) and average relative error (Eq. S12).

$$R^2 = 1 - \frac{\sum_{i=1}^{N}\left(q_{exp} - q_{pred}\right)^2}{\sum_{i=1}^{N}\left(q_{exp} - q_{pred,avg}\right)^2} \quad \text{(S9)}$$

$$R^2_{adj} = 1 - \left[\frac{(1 - R^2) * (N - 1)}{(N - P - 1)}\right] \quad \text{(S10)}$$

$$SSE = \frac{1}{N}\sum_{i=1}^{N}\left(q_{exp} - q_{pred}\right)^2 \quad \text{(S11)}$$

$$ARE = \frac{100}{n} \sum_{i=1}^{n} \left| \frac{q_{exp} - q_{pred}}{q_{exp}} \right| \quad (S12)$$

Where: q_{exp}, q_{pred} and $q_{pred,avg}$ are the experimental, predicted and experimental mean adsorption capacity (mg g^{-1}); *P* is the number of fitted parameters, and *N* is the number of data points.

Cell culture and treatments

The neuron-like cells (SH-SY5Y) were obtained by Rio de Janeiro Cell Bank (RJCB code 0223) and were cultivated under ideal conditions (5% of the CO_2) with Dulbecco's modified Eagle's medium and F12 Medium (DEMEN F12) (Gibco- Sigma-Aldrich) containing 10% of fetal bovine serum (FBS) (Sigma-Aldrich), supplemented with 1% antibiotics penicillin/streptomycin (100 U/ 10 mg mL^{-1}) (Sigma-Aldrich) and l% amphotericin B (250 µg mL^{-1}) (Sigma-Aldrich). SH-SY5Y cells were exposed at concentration curve of the Li_2O-NPS (1 -150µg mL^{-1}) during 24, 48 and 72 h and were realized colorimetric and fluorometric assays to assessment the *in vitro* safety profile of the Li_2O-NPs.

Cellular viability determination

To assess cell viability, the MTT (3-[4,5-dimethylthiazol-2-yl]-2,5 diphenyl tetrazolium bromide) assay was employed. Following a 24/72-hour incubation period, 20 µL of a sterile saline solution containing MTT (5 µg mL^{-1}) was added, and the cells were further incubated for 4 h at 37 ± 2ºC and 5% CO_2. Subsequently, the solution was carefully aspirated, and the formazan crystals were dissolved in 200 µL of dimethylsulfoxide (DMSO). Cell growth inhibition was quantified using a microplate reader (Biochrom

Anthos) at a wavelength (λ) of 570 nm. For the negative control, only cells in culture medium are used and for the positive control, dimethyl sulfoxide (DMSO) was used.

ROS generation

The study conducted tests on Li_2O-NPs at concentrations of 1, 5, 10, 25, 50, 100 and 150 μg mL^{-1} using the 2',7'-dichlorodihydrofluorescein diacetate (DCFH-DA) assay. The DCFH-DA solution (1 mmol L^{-1}) was diluted in ethyl alcohol (1:10 v v^{-1}), and 10 μL of the diluted solution was added to a black plate containing Tris HCl (65 μL) and treated cells (50 μL). For the negative control, only cells in culture medium are used and for the positive control, hydrogen peroxide (H_2O_2) was used. Following a 1-h incubation period, the readings were obtained using a fluorimeter (SpectraMax i3x – Molecular Devices) at 525 nm for emission and 428 nm for excitation wavelengths.

NO generation

In the analysis, Griess solution, consisting of 0.1% N-(1-naphthyl) ethylenediamine dihydrochloride and 1% sulfanilamide in 5% phosphoric acid, was employed to detect the presence of nitrites (NO_2^-). Specifically, 100 μL of the supernatant was combined with 500 μL of Griess solution in a 96-well plate and allowed to incubate for 30 minutes. Subsequently, the intensity of the resultant color at 540 nm was determined using an ELISA reader (photometer). For the negative control, only cells in culture medium are used and for the positive control, nitric oxide (NO) was used.

Determination of dsDNA extracellular release

To quantify the amount of free dsDNA in the extracellular medium, we utilized Quant-iTTM PicoGreen dsDNA. In a 96-well plate, approximately 80 μL of TE 1X (10

mmol L^{-1} Tris-HCl, 1 mmol L^{-1} EDTA) buffer, 10 μL of supernatants, and 10 μL of PicoGreen reagent were combined. Subsequently, the plates were shielded from light and left at 25 ± 2ºC for approximately 5 minutes to allow the reagent to react with the samples. In this experiment, fluorescence was measured at excitation and emission wavelengths of 480 nm and 520 nm, respectively, using a plate reader device (SYNERGY HTX, BioTek). The negative control consisted of cells in culture medium only, while the positive control utilized was hydrogen peroxide (H_2O_2).

Statistical analysis

For the *in vitro* safety profile, the results were statistically analyzed using the GraphPad Prism program, version 8, using one-way ANOVA followed by Tukey's post hoc test. Comparative analyses were considered significant when $p<0.05$, $p<0.05$*, $p<0.01$**, $p < 0.001$*** and $p < 0.0001$****. For CCDR 2^2, Statistic 10 Software (version 10, Statsoft, USA) was used.
